# Design of the HPTN 065 (TLC-Plus) study: A study to evaluate the feasibility of an enhanced test, link-to-care, plus treat approach for HIV prevention in the United States

**DOI:** 10.1177/1740774517711682

**Published:** 2017-06-19

**Authors:** Theresa Gamble, Bernard Branson, Deborah Donnell, H Irene Hall, Georgette King, Blayne Cutler, Shannon Hader, David Burns, Jason Leider, Angela Fulwood Wood, Kevin G. Volpp, Kate Buchacz, Wafaa M El-Sadr, for the HPTN 065 Study Team

**Affiliations:** 1 Science Facilitation Department, HPTN Leadership and Operations Center, FHI 360, Durham, NC, USA; 2 Scientific Affairs, LLC, Atlanta, GA, USA; 3 Vaccine and Infectious Disease Division, Fred Hutchinson Cancer Research Center, Seattle, WA, USA; 4 Division of HIV/AIDS Prevention, National Center for HIV/AIDS, Viral Hepatitis, STD, and TB Prevention, Centers for Disease Control and Prevention, Atlanta, GA, USA; 5 Public Health Foundation Enterprises, La Puente, CA, USA; 6 DC Department of Health, HIV/AIDS, Hepatitis, STD and TB Administration, Washington, DC, USA; 7 Division of AIDS, National Institute of Allergy and Infectious Diseases, Bethesda, MD, USA; 8 Albert Einstein College of Medicine, New York, NY, USA; 9 Family and Medical Counseling Service, Inc., Washington, DC, USA; 10 Center for Health Incentives and Behavioral Economics; Perelman School of Medicine and the Wharton School, University of Pennsylvania, Philadelphia, PA, USA; 11 ICAP at Columbia University, Mailman School of Public Health, New York, NY, USA

**Keywords:** “Test and treat” approach for HIV prevention, expanded HIV testing, linkage to HIV care, HIV viral suppression, financial incentives, pragmatic clinical trial

## Abstract

**Background/Aims:**

HIV continues to be a major public health threat in the United States, and mathematical modeling has demonstrated that the universal effective use of antiretroviral therapy among all HIV-positive individuals (i.e. the “test and treat” approach) has the potential to control HIV. However, to accomplish this, all the steps that define the HIV care continuum must be achieved at high levels, including HIV testing and diagnosis, linkage to and retention in clinical care, antiretroviral medication initiation, and adherence to achieve and maintain viral suppression. The HPTN 065 (**T**est, **L**ink-to-**C**are **P**lus Treat [TLC-Plus]) study was designed to determine the feasibility of the “test and treat” approach in the United States.

**Methods:**

HPTN 065 was conducted in two intervention communities, Bronx, NY, and Washington, DC, along with four non-intervention communities, Chicago, IL; Houston, TX; Miami, FL; and Philadelphia, PA. The study consisted of five components: (1) exploring the feasibility of expanded HIV testing via social mobilization and the universal offer of testing in hospital settings, (2) evaluating the effectiveness of financial incentives to increase linkage to care, (3) evaluating the effectiveness of financial incentives to increase viral suppression, (4) evaluating the effectiveness of a computer-delivered intervention to decrease risk behavior in HIV-positive patients in healthcare settings, and (5) administering provider and patient surveys to assess knowledge and attitudes regarding the use of antiretroviral therapy for prevention and the use of financial incentives to improve health outcomes. The study used observational cohorts, cluster and individual randomization, and made novel use of the existing national HIV surveillance data infrastructure. All components were developed with input from a community advisory board, and pragmatic methods were used to implement and assess the outcomes for each study component.

**Results:**

A total of 76 sites in Washington, DC, and the Bronx, NY, participated in the study: 37 HIV test sites, including 16 hospitals, and 39 HIV care sites. Between September 2010 and December 2014, all study components were successfully implemented at these sites and resulted in valid outcomes. Our pragmatic approach to the study design, implementation, and the assessment of study outcomes allowed the study to be conducted within established programmatic structures and processes. In addition, it was successfully layered on the ongoing standard of care and existing data infrastructure without disrupting health services.

**Conclusion:**

The HPTN 065 study demonstrated the feasibility of implementing and evaluating a multi-component “test and treat” trial that included a large number of community sites and involved pragmatic approaches to study implementation and evaluation.

## Introduction

HIV continues to be a major public health threat in the United States. Approximately 1.2 million people above the age of 12 years are living with HIV in the United States and about 40,000 infections are diagnosed each year.^1,2^ The efficacy of antiretroviral therapy for preventing HIV transmission was confirmed by HPTN 052^3^ and its role in reducing HIV incidence is supported by ecological^4–6^ and observational^7–10^ studies, in addition to mathematical modeling.^11–21^ However, to achieve this potential, all the steps that define the HIV care continuum must be achieved at high levels, including HIV testing and diagnosis, linkage to and retention in clinical care, antiretroviral medication initiation, and adherence to achieve and maintain viral suppression.^22,23^ The HPTN 065 (**T**est, **L**ink-to-**C**are **P**lus Treat [TLC-Plus]) study was designed as a community-based trial with interventions that parallel the key steps of the HIV care continuum. Our goal was to optimize the outcomes at each of these steps to determine the feasibility of the “test and treat” approach in US communities.

## Rationale for study components

**Figure 1.**
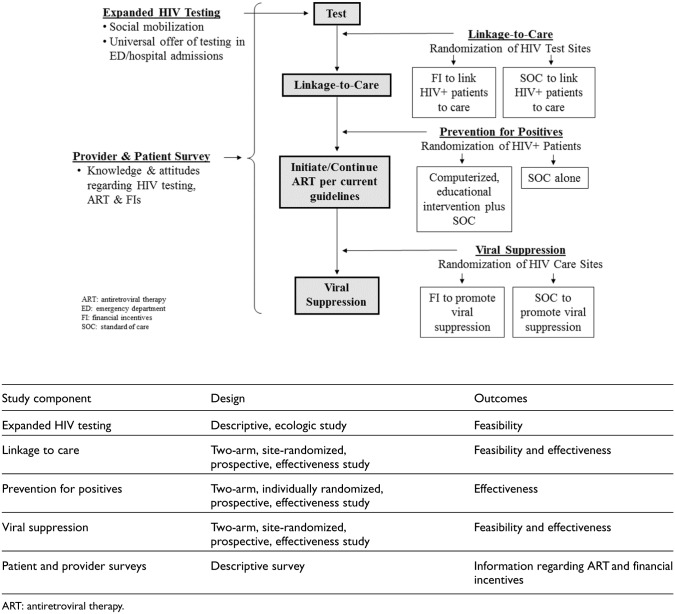
HPTN 065 study components.

HPTN 065 consisted of five components: (1) exploring the feasibility of expanded HIV testing via social mobilization and the universal offer of testing in hospital settings, (2) evaluating the effectiveness of financial incentives to increase linkage to care, (3) evaluating the effectiveness of financial incentives to increase viral suppression, (4) evaluating the effectiveness of a computer-delivered intervention to decrease risk behaviors in HIV-positive patients, and (5) administering provider and patient surveys to assess knowledge and attitudes regarding the use of antiretroviral therapy for prevention and the use of financial incentives to improve health outcomes (Figure 1).

In the expanded HIV testing component, the study used two interventions: (1) social mobilization to increase HIV testing among men who have sex with men (MSM) and (2) expanded HIV testing in emergency departments and inpatient units at participating hospitals. The study elected to encourage MSM to test more frequently given disproportionate risk in this population. Expanding HIV testing in hospital settings was based on the 2006 Centers for Disease Control and Prevention’s (CDC) recommendation for routine opt-out HIV screening of patients aged 13–64 years in healthcare settings, including hospital emergency departments and inpatient services.^24^

Linkage to care after testing positive for HIV infection is a critical element in the HIV care continuum and is essential for success of the “test-and-treat” approach.^23^ Interventions to improve linkage have included linkage case management,^25,26^ intensive outreach,^27,28^ and peer navigation.^29^ In the United States, only 75% of individuals link to care within 1 month of their diagnosis.^1^ Viral suppression offers clinical benefit to the individual and significantly decreases the risk of HIV transmission to uninfected sex partners.^3^ However, only 55% of persons living with HIV in the United States achieve viral suppression.^30^ Several factors reduce adherence to treatment, and, accordingly, several interventions to enhance adherence have been assessed.^31,32^

Financial incentives appear encouraging based on their success in the adoption of positive health behaviors (e.g. smoking cessation),^33^ as well as HIV-related behaviors such as HIV testing^34–36^ and antiretroviral pill-taking.^37–40^ A small study conducted in India demonstrated improvements in linkage to care among 120 HIV-positive drug users when financial incentives were offered^41^ and another study of 90 non-adherent patients in the United States demonstrated an association between financial incentives and reductions in viral load suppression.^42^ However, a larger study of 801 HIV-positive substance users found no improvements in viral load suppression with the use of financial incentives.^43^ We chose to test whether financial incentives could increase linkage to care and viral suppression because financial incentive interventions could be implemented at scale without additional skilled personnel or disruption to normal clinic flow; and, because they have been successful in changing both HIV and other health-related behaviors.

Adoption and maintenance of safer behaviors by HIV-positive persons is important for reducing HIV transmission. While data indicate that persons with HIV adopt safer behaviors once they are aware of their infection,^44^ maintaining safer behaviors is challenging with HIV-positive individuals reporting sex without condoms.^45^ Approaches for prevention among HIV-positive persons (referred to as “prevention for positives”) have included both individual and group interactions facilitated by HIV care providers, case managers, health educators, therapists, and HIV-positive peers.^46^ However, many of these interventions require substantial investments of staff time and other resources. A meta-analysis in HIV-negative individuals has shown computer-based interventions to be efficacious in increasing self-reported condom use, reducing the number of sex partners, and decreasing the incidence of sexually transmitted diseases.^47^ Such computer-based interventions have also successfully reduced self-reported transmission risk behavior in those who are HIV-positive.^48,49^ This type of intervention offers several potential advantages: utilization of patient time while awaiting consultation, delivery of customized content with fidelity, capacity to use multiple languages, and lowered costs. CARE+, the intervention adapted for and assessed in HPTN 065, is a multimedia, audio-narrated, interactive health communication tool designed to assess HIV risk behavior and, based on user responses, provide tailored feedback, behavioral skill-building videos, and development of a specific and realistic risk-reduction plan.^50^ It is acceptable to both patients and providers^50^ and is effective in reducing self-reported sexual transmission risk behaviors.^48^

The final component of HPTN 065 was surveys, conducted with both providers and patients, that assessed acceptability, knowledge, attitudes, and practices with regard to antiretroviral therapy initiation, the use of antiretroviral therapy for the prevention of HIV transmission, and the use of financial incentives to motivate linkage to care and viral suppression.^51,52^ This last component was important because provider and patient buy-in to the principles of the “test and treat” approach is essential for its success.

## Study methods

Study methods are summarized below, and access to the complete protocol is provided at the HPTN website.^53^

### Community engagement

An HPTN 065 community advisory group was formed as the study was being designed. It consisted of 17 members who were engaged and knowledgeable about HIV advocacy at the site, community, and national level. The community advisory group provided recommendations for the design of the financial incentive interventions, the amount of the financial incentives for each component, participant-related procedures, and the provider and patient surveys. In addition, the group reviewed presentation and training materials and was engaged in the dissemination of study results to the community.

### Study communities and sites

The study included six communities in the United States: two intervention and four non-intervention. The Bronx, NY, and Washington, DC, were chosen for the study interventions because they represented geographic areas with high rates of HIV diagnosis and prevalence; had already undertaken substantial efforts to improve HIV testing, linkage to care and adherence; and maintained robust HIV surveillance systems. HIV test and care facilities in these two intervention communities were selected based on the following criteria: (1) HIV test sites with the largest number of HIV-positive individuals identified in the previous year, (2) HIV care sites with the largest number of HIV-positive patients in care in the previous year, and (3) site agreement to study participation, including randomization. The process of site selection has been previously described.^54^ Four communities (Chicago, IL; Houston, TX; Miami, FL; and Philadelphia, PA) were chosen as non-intervention communities. These communities were selected for similar reasons as the intervention communities and were included to assess secular trends in HIV testing, linkage to care and viral suppression over the time period of the study interventions. Assessments of non-intervention communities used data from an annual survey that collected information from each local department of health and the US National HIV Surveillance System.

### Ethical review and informed consent

The study used both central and local Institutional Review Boards (IRBs) to obtain ethical review and approval for study conduct. For three of the study components (expanded HIV testing in hospital settings and both financial incentive interventions), we sought and obtained waivers of consent under 45 CFR 46.116 (d) because the interventions were of minimal risk, a waiver of informed consent would not adversely affect the rights or welfare of the participants, and these study components could not have been practicably carried out without a waiver. The IRBs were also informed that the outcomes were reported using either site-aggregated surveillance data (financial incentive interventions), or site-aggregated testing data (expanded HIV testing in hospital settings), with no individual data collected from the sites. Individual consent was obtained for the prevention for positives and the patient and provider survey components.

### Social mobilization for expanded HIV testing

HPTN 065 built on the existing HIV testing efforts in both intervention communities. In the Bronx, the study team worked with the New York City Department of Health and Mental Hygiene to create additional materials targeting MSM for *The Bronx Knows*^55^ campaign. In Washington, DC, the study team worked with the Washington DC Department of Health to create additional materials urging MSM to get tested for HIV twice a year as part of the *Ask for the Test*^56^ campaign. The additional materials were disseminated via posters, bus advertisements, local newspapers, radio spots, promotional materials, and websites. In the Bronx, NY, the New York City Department of Health and Mental Hygiene also created and disseminated a campaign called *Say Yes to the Test*. The purpose of this additional campaign was to remind patients of a new 2010 law that required healthcare providers in NY to offer a voluntary HIV test to all patients aged 13–64 years and to encourage acceptance of the offer.^57^ The public was exposed to these enhanced campaigns over the course of 3 years.

The study used local behavioral surveys to compare the level and outcomes of HIV testing, as well as to assess the frequency and costs of the social mobilization activities.

### Expanded HIV testing in the emergency department and inpatient settings

The study encouraged participating hospitals to move from point-of-care HIV rapid testing to lab-based testing with rapid turnaround for results as a way to achieve higher testing volumes and detect infection sooner after exposure with less cost.^58^ However, recognizing the diversity of settings and practices, all participating hospitals were asked to develop their own strategies for increasing HIV testing and could use their study funds as they saw fit. The hospitals’ strategies were documented throughout the 3-year period of implementation.

Outcome measures collected from each hospital included the number and proportion of emergency department visits and inpatient admissions where HIV testing was done, the results of these tests, and the costs associated with increased testing. Process measures for expanded HIV tested included (1) administrative and staff changes to support expanded HIV testing, (2) structural changes, such as including consent for HIV testing in the general hospital consent for care, and (3) laboratory-related changes, such as acquisition and use of multiplatform automated analyzers for HIV testing.

### Financial incentives for linkage to care

Participating HIV test sites were randomized by site (cluster randomization) to either the financial incentive or standard of care arm for the linkage to care component of the study. Each test site completed an annual survey documenting their standard of care procedures for HIV testing and linking individuals to care.

HIV test sites assigned to the financial incentive arm distributed coupons to newly diagnosed HIV-positive individuals and those previously diagnosed, but out of HIV care for more than 1 year. Patients could redeem linkage coupons for gift cards when they sought HIV care within 3 months at any of the participating HIV care sites: a US$25 gift card when they had blood drawn for viral load and CD4 testing and a US$100 gift card after completing a visit with a clinical provider to develop a plan for HIV medical care. The linkage to care component was conducted for a period of 2 years.

The study planned to use 40 sites (20 per arm). Based on previous estimates of the average number of new HIV-diagnoses and linkage rates derived from HIV surveillance data, we estimated we would have 80% power to detect an absolute 13% increase in the proportion linked to care (comparing financial incentive to standard of care sites).

### Financial incentives for viral suppression

Participating HIV care sites were randomized by site to the financial incentive or standard of care arm. Site randomization was balanced based on two baseline characteristics: (1) the number of HIV-positive patients in care and (2) the proportion of HIV-positive patients with viral load suppression. Balance was achieved by selecting at random from the 1000 randomizations with the smallest absolute difference between-arm t-test statistics, using existing baseline data from the HIV surveillance system.^54^

At sites assigned to the financial incentive intervention, patients who were established in care (defined as having a viral load measurement at the site within the last 3 to 9 months) and on treatment were eligible to receive financial incentives. Patients received a US$70 gift card every 3 months if they had a suppressed viral load (defined as HIV RNA < 400 copies/mL). The financial incentive intervention for viral suppression was conducted for a period of 2 years. Each participating care site completed an annual survey documenting their standard of care procedures for helping patients achieve and maintain viral suppression. The study planned to use 40 sites (20 per arm). Based on estimates from the surveillance system of the average number of patients at sites and the proportion virally suppressed, we estimated the study would have 90% power to detect a 6% absolute increase in the proportion virally suppressed (comparing financial incentive to standard of care sites).

### Measuring outcomes of linkage to care and viral suppression study components

A unique feature of HPTN 065 was the use of the US National HIV Surveillance System to determine the primary effectiveness outcomes for both financial incentive interventions. Laws in each jurisdiction mandate laboratories to report, by name, positive HIV test results, HIV viral load values, and CD4 T-cell counts.^59^ To maintain the confidentiality of the surveillance data, only de-identified site-aggregate data were used for analysis as described previously.^54^

Linkage to care was assessed for each site as the percentage of newly HIV-diagnosed and out-of-care patients who had a viral load or CD4 measurement within 3 months of their positive HIV test result. Viral suppression was assessed for each site as the proportion of established patients with a suppressed viral load within each quarter; patients were considered established in care if they had HIV laboratory tests in two different calendar quarters over the prior 15 months at that site. For both of these cluster randomized components, the site-aggregate outcomes were used to compare average responses between the arms, appropriately adjusted for cluster size and baseline measures.

The study also monitored the provision of financial incentives for viral suppression by tracking the proportion and number of patients who were eligible for and received US$70 gift cards. To determine whether patients were switching from sites that did not offer financial incentives for viral suppression to sites that did, we monitored changes in the size of the patient population at each site throughout the study.

### Prevention for positives

The primary objective of the prevention for positives study component was to evaluate the effectiveness of CARE+ on the number of unprotected sex acts, the number of sexual partners, and needle-sharing behavior. Sites in the prevention for positives study component were selected based on the number of HIV-positive patients in care and the site’s willingness to participate. HIV-positive patients were consented individually and randomized to either the CARE+ intervention or the control arm. The CARE+ intervention was administered via a computer tablet and headphones. In both arms, participants used the computer tablet to undergo a self-administered risk assessment. In the intervention arm, participants also used the computer table to receive the intervention, which included behavioral skill-building videos and receipt of an individualized risk-reduction plan. The target study enrollment for this study component was 1320 individuals. All patients in this component participated in the study for 12 months. Assuming 1223 persons completed all four assessments with an intra-person correlation coefficient of 0.3, the study had >90% power to detect a decrease from 5.4% to 4% in the proportion of patients reporting any unprotected vaginal or anal sex within the 3 months prior to a given study visit.

### Provider and patient surveys

The patient survey was incorporated into the CARE+ software and administered to all participants enrolled into the prevention for positives study component at baseline and the last (month 12) study visit. The provider survey was administered before and after implementation of the financial incentive intervention for viral suppression to assess trends in provider knowledge, attitudes, and practices. Clinical providers who prescribed antiretroviral therapy (e.g. physicians, nurse practitioners, and physician assistants) at all participating HIV care sites in the Bronx, NY, and Washington, DC, were invited to complete the online survey. Descriptive statistics were used to summarize the data from both patients and providers.

## Results

HPTN 065 was conducted between September 2010 and December 2014 with the time frame of each component as follows: expanding HIV testing (November 2010 to January 2014), evaluating financial incentives to increase linkage to care (April 2011 to December 2012), evaluating financial incentives to increase viral suppression (February 2011 to January 2013), evaluating the prevention for positives intervention (January 2013 to December 2014), and administering provider and patient surveys (September 2010 to December 2014).

A total of 76 sites (37 HIV test sites and 39 HIV care sites) were chosen in the intervention communities, representing a wide diversity of test and care facilities (Table 1). The 37 HIV test sites included the 16 hospitals (9 in the Bronx, NY, and 7 in Washington, DC) where efforts to expand HIV testing in admissions to emergency departments and inpatient units took place. Using cluster randomization, 19 HIV test sites were assigned to the financial incentive arm and 18 were assigned to the standard of care arm for the linkage to care component. Two university-affiliated hospital test sites in the Bronx were randomized as one entity because the surveillance data, through which the primary effectiveness outcome was assessed, could not differentiate the two sites operated by the same institution (Table 2). Using a second cluster randomization, 19 HIV care sites were assigned to the financial incentive arm and 20 were assigned to the standard of care arm for the viral suppression component.

**Table 1.** Characteristics of HIV test and care sites in the Bronx, NY, and Washington, DC.

Type of site	Bronx test	Bronx care	DC test	DC care	Total
Community health center/clinic	7 (39%)	11 (55%)	8 (42%)	8 (42%)	34 (45%)
Hospital (non-university affiliated)	4 (22%)	4 (20%)	3 (16%)	3 (16%)	14 (18%)
University-affiliated hospital/clinic	3 (16%)^a^	3 (15%)	3 (16%)	2 (11%)	11 (14%)
Community-based organization	2 (11%)	0 (0%)	3 (16%)	0 (0%)	5 (7%)
Private medical practice	0 (0%)	0 (0%)	0 (0%)	5 (26%)	5 (7%)
VA facility^b^	1 (6%)	1 (5%)	1 (5%)	1 (5%)	4 (5%)
STI clinic	1 (6%)	0 (0%)	1 (5%)	0 (0%)	2 (3%)
Substance abuse clinic	0 (0%)	1 (5%)	0 (0%)	0 (0%)	1 (1%)
Total	18	20	19	19	76

DC: Washington, DC; STI: sexually transmitted infections; VA: Veterans Affairs.

a Two university-affiliated hospital test sites were randomized as one entity because their surveillance data could not be differentiated between the two related facilities. However, they were treated as unique entities for the expanded HIV testing in hospital settings study component.

b Both VA facilities included a hospital and conducted expanded HIV testing activities.

**Table 2.** Site randomization for linkage to care and viral suppression study components.

HPTN 065 sites	DC		Bronx		Total	
	Test	Care	Test	Care	Test	Care
All sites	19	19	18	20	37	39
L2C–FI	10		9		37	
L2C–SOC	9		9			
VS–FI		9		10		39
VS–SOC		10		10		
Hospitals	7		9^a^		16	
Site surveys	19	19	18	20	37	39
PfP		6		5		11

FI: financial incentive; L2C: linkage to care; PfP: prevention for positives; SOC: standard of care.

Institutional Review Board (IRB) review and approval was sought and obtained for every participating site either at the IRB affiliated with the site or via a commercial IRB.

a Two university-affiliated hospital test sites in the Bronx were randomized as one entity because the surveillance data could not be differentiated between the two related facilities. Therefore, they were asked to complete a single site survey. However, these hospitals were treated as unique entities for the expanded HIV testing in hospital settings study component.

Out of the 39 HIV care sites, 11 participated in the prevention for positives study component, six in Washington, DC, and five in the Bronx, NY, with approximately half in each city in each arm of the viral suppression component (to mitigate potential for interaction). At these sites, 1075 participants were enrolled and randomized to either the intervention or a control arm. These same participants were invited to complete the patient surveys. For the provider surveys, there were a total of 165 respondents at baseline and 141 respondents at follow-up.

Data collected from the non-intervention communities will be reviewed in the context of changes in the surveillance data for the same outcomes as the intervention communities.

## Discussion

The HPTN 065 study was designed to determine the feasibility of implementing various interventions along the HIV care continuum to achieve the “test and treat” strategy. To accomplish this, we implemented large-scale interventions with the potential to affect outcomes at a community level. All study interventions were selected to be focused, adaptable, and feasible in clinical settings without substantial additional resources for their implementation or the assessment of their outcomes. We were able to successfully implement all of the study components and achieve valid outcomes, demonstrating feasibility of this multilayered, pragmatic approach.

In general, we found it feasible to conduct study interventions within the usual structures and procedures at participating sites. This was achieved by taking into account their normal processes and largely depending on their programmatic staff. The study included a wide variety of sites, which were allowed substantial flexibility to adapt study implementation for their individual context. This approach was intended to enhance generalizability of our study findings to disparate settings; however, it also had implications for study outcomes. As one example, some test sites were co-located with HIV care sites, while others were geographically distinct. These differences likely influenced the process of linkage and the way the financial incentive intervention could be implemented at different sites. As another, there was a change in the US HIV treatment guidelines during the conduct of the study, from a recommendation to initiate antiretroviral therapy in persons with CD4 < 500^60^ to one of treatment for all HIV-positive individuals irrespective of CD4 cell count.^61^ However, while the study provided information regarding such a change to all HIV care sites, it did not require specific treatment approaches or monitor provider practices; thus, there may have been variability in treatment strategies by provider and site.

In keeping with the desire to work within the existing healthcare system and without additional staff and patient burden, the study aimed to use data that were already being collected, rather than collecting data separately via usual research methods for the study. However, this resulted in some challenges. For example, routinely collected HIV testing data were used to assess the expanded HIV testing efforts in the hospital settings; unfortunately, these data were often incomplete or were not collected uniformly at all hospitals. Another example was our novel use of HIV confirmatory test results, CD4 counts, and viral load values reported by laboratories to the US National HIV Surveillance System to assess the primary outcomes of the financial incentive interventions on linkage to care and viral suppression. However, surveillance data did not allow us to ascertain whether patients were on antiretroviral therapy, and our use of it was complicated by the fact that some laboratory data were reported to surveillance in the jurisdiction of the patient’s place of residence, rather than to the one in which they received care. In addition, the robustness of the surveillance data varied by jurisdiction in terms of data completeness and timeliness. Resolving these issues required intense investments of effort to improve data quality to an acceptable level.

The use of cluster randomization of the HIV test sites and HIV care sites for the financial incentive interventions presented both advantages and challenges for implementation. This site-randomized design avoided the logistical difficulties that might occur if incentives were offered to some, but not all, individuals who qualified for incentives at a given HIV test or care site. However, participating sites initially raised concerns that patients at sites randomized to the standard of care arm might attempt to migrate to financial incentive sites, potentially overwhelming some sites with new patients or depleting the patient population of other sites. Several study procedures were put in place to minimize such migration. For example, patients had to be established in care for at least 3 months before they were eligible to receive financial incentives for viral suppression. Thus, patients who switched providers just to receive financial incentives would have had to wait a substantial amount of time before they qualified for the incentive. Similarly, testing sites were instructed not to give more than one coupon to any individual, care sites were instructed not to redeem more than one coupon for any individual, and the coupons were created using uniquely colored and embossed paper to make them difficult to reproduce.

The use of multiple randomization schemes within the study could have potentially interfered with study outcomes. The individual randomization for the prevention of positives component occurred at sites that were also part of the site-randomization for financial incentives for viral suppression. However, while both cluster and individual randomization were used in different components of the study, each component was completely independent. Sites for linkage to care and viral suppression, while both involved in cluster randomizations, did not involve the same locations. Moreover, the outcomes of the financial incentive interventions were at different, distinct stages of the treatment continuum so could not have involved the same individuals at the same time. Finally, enrollment of individuals into the prevention for positives component began as the financial incentive intervention for viral suppression was ending; thus, explicit interactions between the two interventions were not possible.

In summary, HPTN 065 was an ambitious 4-year multi-component study that aimed to assess the feasibility of a “test and treat” approach for HIV prevention in the United States at a community level. The two study intervention communities, the Bronx, NY, and Washington, DC, allowed for the results to be generalizable to other urban communities with substantial rates of HIV. The study’s design was successful in using approaches that fit within established structures and that could be layered on the ongoing standard of care without disrupting health services. This study demonstrated the possibility of implementing and evaluating “test and treat” strategies if pragmatic and community-focused approaches are used.
